# Real-Time Reflectance Measurement Using an Astigmatic Optical Profilometer

**DOI:** 10.3390/s22166242

**Published:** 2022-08-19

**Authors:** Hsien-Shun Liao, Ya-Kang Huang, Jian-Yuan Syu-Gu, En-Te Hwu

**Affiliations:** 1 Department of Mechanical Engineering, National Taiwan University, Taipei 10617, Taiwan; 2 Department of Health Technology, Technical University of Denmark, 2800 Lyngby, Denmark

**Keywords:** reflectance, astigmatism, optical profilometer

## Abstract

An astigmatic optical profilometer with a commercial optical pickup head provides benefits, such as high resolution, compact size, and low cost. To eliminate artifacts caused by complex materials with different reflectances, a z-axis modulation mode is proposed to obtain quantitative surface morphology by measuring S curves on all image pixels. Moreover, the slope of the linear region in the S curve shows a positive relationship with the surface reflectance. However, the slope was calculated using an offline curve fitting method, which did not allow real-time reflectance imaging. Furthermore, quantitative reflectance data were unavailable because of the lack of calibration. In this study, we propose a novel method for real-time reflectance imaging by measuring the amplitude of a focus error signal (FES). The calibration results displayed a linear relationship between the FES amplitude and reflectance. The reflectance image of a grating sample with chrome patterns on a glass substrate demonstrates accurate reflectance measurements with a micrometer spatial resolution.

## 1. Introduction

Optical reflectance measurement has a wide range of applications, including studying the optical adsorption of polymers, investigating the optical properties of surface coatings, and analyzing the optical characteristics of biological tissues [1,2,3,4]. For diverse needs, different instruments, such as an integrating sphere reflectometer, laser polarimeter, and glossmeter, have been used to measure the reflectance [5,6,7,8]. Surface reflectance can be significantly influenced by microroughness, microstructures, and complex materials [9,10,11]. However, the spatial resolution of common reflectometers is usually in the millimeter scale, owing to the beam spot size of the light source [12,13].

By adopting a commercial digital versatile disk (DVD) pickup head, the astigmatic profilometer is equipped with the advantages of small beam spot size, compact size, and high bandwidth [14,15,16]. For sensing small data pits, the focal laser spot on the DVD pickup head has a small full width at half maximum (FWHM) of 530 nm [17]. Therefore, the astigmatic profilometer is able to provide a high spatial resolution image, which has been utilized for various applications, such as bio-molecular sensing and cell imaging [18,19,20]. Previous studies have demonstrated that the astigmatic profilometer can measure not only the surface morphology but also the film thickness [21]. Moreover, the z-axis modulation mode of the astigmatic profilometer enables measurement of the S curve at each image pixel [22,23]. The slope of the S-curve linear region was positively correlated with the surface reflectance. However, the quantitative relationship between the slope and reflectance has not yet been identified. Moreover, fast S-curve measurement requires high-speed data acquisition with a megahertz sampling rate and gigabyte data transfer from the controller to a personal computer. Furthermore, slope calculation requires a curve-fitting algorithm, which is not convenient for implementation in the field-programmable gate array (FPGA) of the controller. Therefore, the slope was calculated offline using MATLAB software on a personal computer.

In this study, a method for real-time reflectance imaging was demonstrated. In this method, the objective lens of a DVD pickup head is oscillated vertically using a resonant scanner to generate the focus error signal (FES) oscillation. The FES amplitude signal can be directly measured by the controller by integrating a lock-in amplifier into an astigmatic profilometer. After calibration, the FES amplitude signal was directly converted into reflectance for real-time imaging. The experimental results prove that the proposed method can provide accurate reflectance images and further expand the application field of astigmatic profilometers.

## 2. Materials and Methods

### 2.1. Method of Real-Time Reflectance Measurement

**Figure 1:**
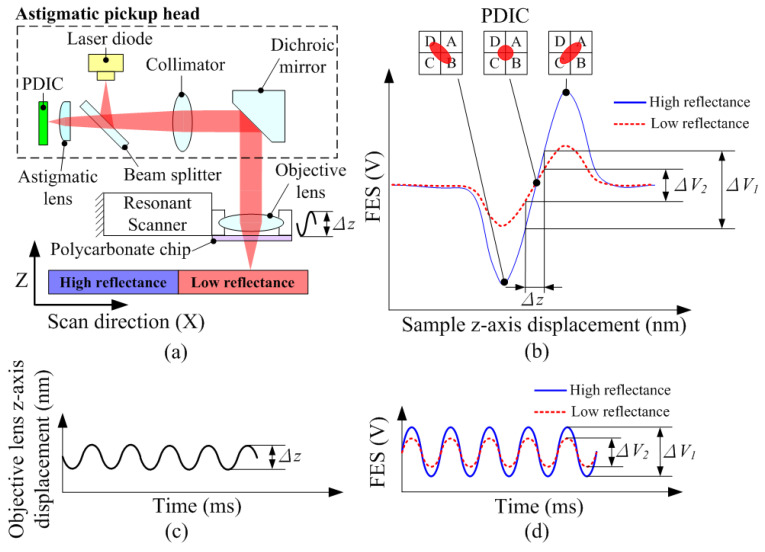
(**a**) Optical configuration for the reflectance measurement. (**b**) FES vs. sample z-axis displacement curves with different surface reflectances. (**c**) objective lens z-axis displacement vs. time curve. (**d**) FES vs. time curves with different surface reflectances.

Figure 1a illustrates the proposed optical configuration for reflectance measurement based on an astigmatic pickup head. In the pickup head, the laser beam from the laser diode is reflected by a beam splitter and collimated through a collimator lens. A dichroic mirror reflected the horizontal red-light beam downward into an objective lens to focus it on the sample surface. A polycarbonate chip was glued below the objective lens to optimize measurement sensitivity [24]. Moreover, an optical microscope can be used to monitor the measurement position on the sample surface through a dichroic mirror [25]. After passing through the beam splitter and astigmatic lens, the return beam was detected using a photodetector-integrated chip (PDIC). As illustrated in Figure 1b, the z-axis displacement of the sample caused a shape variation in the laser spot on the PDIC owing to astigmatism, which can be detected by the FES defined in (1):

(1) $$\mathrm{FES}=(S_{A}+S_{C})-(S_{B}+S_{D})$$

where *S_A_*, *S_B_*, *S_C_*, and *S_D_* represent the voltage signals generated from the four-quadrant photosensors A, B, C, and D, respectively. The relationship curve between the FES and the z-axis displacement of the sample is called the S curve. In the linear region of the S curve, the FES is proportional to the z-axis displacement of the sample and can, therefore, be used to measure the surface morphology by scanning the sample in the XY plane. Moreover, the reflectance of the sample surface affects the laser intensity of the PDIC. The solid blue line and dotted red line in Figure 1b illustrate the S curves on the high- and low-reflectance surfaces, respectively. The slope of the linear region of the S curve on the high-reflectance surface was higher than that on the low-reflectance surface. To realize real-time reflectance imaging, the objective lens was removed from the original pickup head and installed on a resonant scanner to generate a fast z-axis oscillation Δ*z*, as shown in Figure 1c. Owing to the change in the relative displacement between the objective lens and the sample, oscillation Δ*z* can also generate FES variation. Figure 1d shows the oscillations Δ*V*_1_ and Δ*V*_2_ on high- and low-reflectance surfaces, respectively. The amplitude of the FES oscillation is proportional to the slope of the linear region and can be converted into a voltage signal by utilizing a lock-in amplifier or an RMS-to-DC converter.

### 2.2. Astigmatic Optical Profilometer

**Figure 2:**
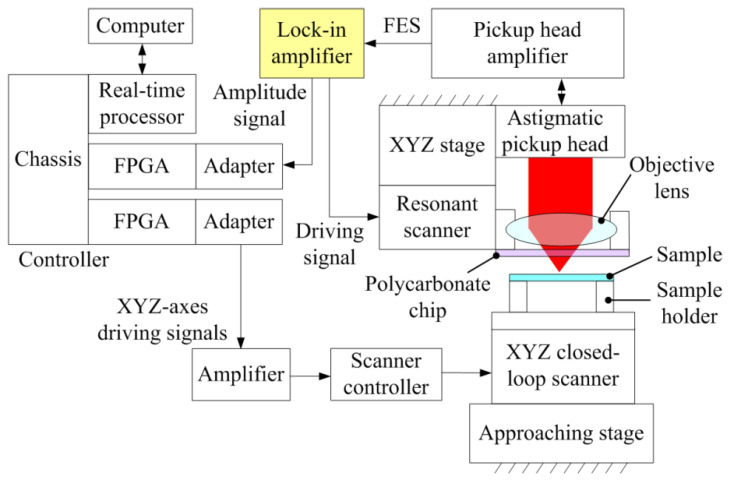
System configuration of an astigmatic optical profilometer for real-time reflectance imaging.

Figure 2 shows the configuration of the astigmatic optical profilometer used for real-time reflectance imaging. A commercial DVD pickup head (TOP1100s, TopRay Technologies, Hsinchu City, Taiwan) was installed on a manual XYZ stage (XC2-60SMW and ZA-60MBW, Twin Nines, Saitama, Japan) to adjust the measurement location of the sample. We used a previously designed resonant scanner to oscillate the objective lens in the z-axis direction [23]. The resonant scanner was operated at a resonant frequency of 1.6 kHz, which can achieve a travel range of >87 μm. For reflectance measurement, only a small oscillation of hundreds of nanometers is required. Therefore, the piezoelectric actuators of the resonant scanner can be directly driven by a lock-in amplifier (SR830, Stanford Research Systems, Sunnyvale, CA, USA) without a high-voltage amplifier. The lock-in amplifier also received the FES from a homemade pickup head amplifier and generated an FES amplitude signal. The FES amplitude signal was captured by a programmable controller (PXIe system, National Instruments, Austin, TX, USA) equipped with a chassis (PXIe-1062Q), a real-time controller (PXIe-8840), and two FPGA modules (PXIe-7961R) with adaptors (NI-5781). The programmable controller was also responsible for generating XYZ-axis control signals for a closed-loop piezoelectric scanner (P-611.3S NanoCube, Physik Instrumente, Karlsruhe, Germany) with a travel range of 100 μm in the XYZ direction. Because the output voltage range of the adaptors was only −1 to 1 V, an amplifier made in house was utilized to magnify the control signals to fit the input range of the scanner controller (E-664. S3 Piezo Controller, Physik Instrumente, Karlsruhe, Germany). The Z-axis displacement of the closed-loop scanner was only used to measure the S curve for calibration, which is not essential for reflectance imaging. A manual approaching stage was placed under the closed-loop scanner to bring the sample into the measurable range of the pickup head.

## 3. Results and Discussion

### 3.1. S-Curve Slope vs. Reflectance

**Figure 3:**
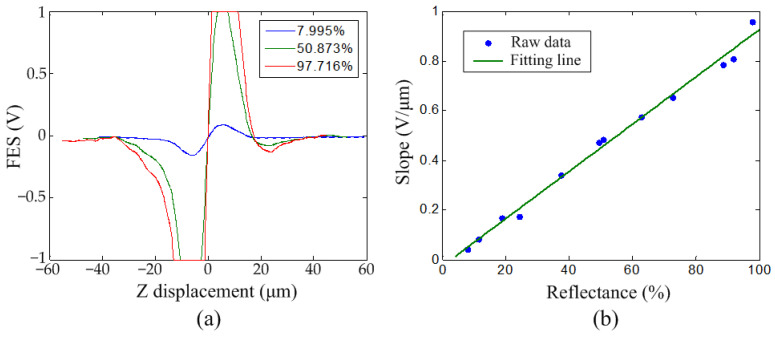
(**a**) FES vs. Z-axis displacement curves and (**b**) relationship between the slope of linear region and reflectance for optical filters with varying reflectances.

Twelve optical filters (ROCOES Electro-Optics, Taichung, Taiwan) with different reflectance values were used for calibration. Table 1 shows the reflectances of the filters, which ranged from 7.995% to 97.716% when measured using a commercial reflection measurement system (MFS-R, Hong-Ming Technology, New Taipei City, Taiwan). The S curves on the filters were first measured to examine the relationship between the slope in the linear region and the reflectance. In this experiment, the filter sample was manually adjusted to a vertical position around the focal plane of the pickup head. The sample was then moved vertically by the closed-loop scanner at a constant velocity and the FES was recorded during the movement. Figure 3a shows the S curves for three selected filters, with reflectances of 7.995%, 50.873%, and 97.716%, respectively. The slope increased with the reflectance. The FES was saturated at −1 and 1 V, owing to the input voltage range of the adaptors. The slope in the linear region of each S curve was calculated by linear curve fitting using MATLAB software. Figure 3b shows the relationship between slope and reflectance. The blue dots and green line represent the raw data and fitting line, respectively. The results show that the slope is proportional to the reflectance and the fitting line is linear with a coefficient of determination, *R*^2^, of 0.9916. The equation of the fitting line is shown in (2):

(2) Slope (V/μm)=0.009501 × Reflectance (%)−0.02388

### 3.2. Amplitude Signal vs. Reflectance

**Figure 4:**
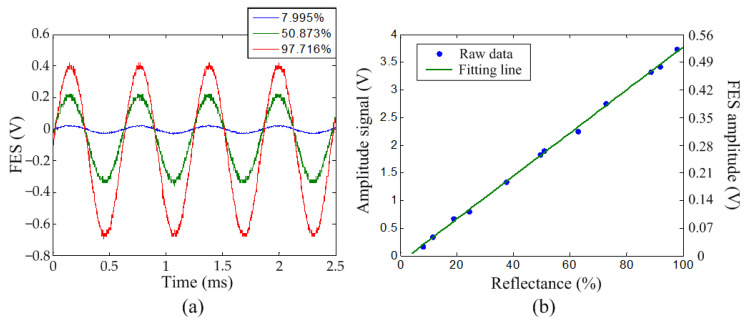
(**a**) FES vs. time and (**b**) relationship between the amplitude signal and reflectance for optical filters with different reflectances.

Because the slope was not available during real-time imaging, the relationship between the amplitude signal and reflectance was also calibrated. In this experiment, the resonant scanner was driven by the lock-in amplifier using a sinusoidal waveform with a driving amplitude of 128 mV at 1.625 kHz. The amplitude of displacement was 546 nm, which was calibrated using the 99.716% reflectance filter with a known slope, as shown in Figure 3b. A large amplitude can enhance the signal-to-noise ratio of the amplitude signal for improving the reflectance resolution. However, the FES needs to be kept in the linear region of the S curve to maintain the measurement accuracy. The vertical position of the sample was manually adjusted to the focal plane of the pickup head. Figure 4a displays the FES vs. time diagram for the three selected filters, with reflectances of 7.995%, 50.873%, and 97.716%, respectively. The FES on the higher-reflectance filter clearly showed a larger amplitude than that on the lower-reflectance filter. The amplitude signal was generated by the lock-in amplifier using a time constant of 3 ms and a full-scale sensitivity of 1 V. There is a trade-off between the reflectance sensitivity and the measurable reflectance range. Using a smaller full-scale sensitivity (higher gain) can obtain a higher reflectance sensitivity, but signal overload in the lock-in amplifier needs to be prevented on the high-reflectance surface. Figure 4b shows the relationship between the amplitude signal and reflectance. The amplitude signal can be converted into the actual FES voltage amplitude by multiplying it by a constant of 0.14, as shown on the right vertical axis in Figure 4b. Similar to the result in Figure 3b, the relationship between the amplitude signal and reflectance was highly linear, with an *R*^2^ of 0.9989, and the linear fitting line is expressed in (3):

(3) Amplitude signal (V)=0.03885 × Reflectance (%)−0.1089

### 3.3. Real-Time Reflectance Imaging

**Figure 5:**
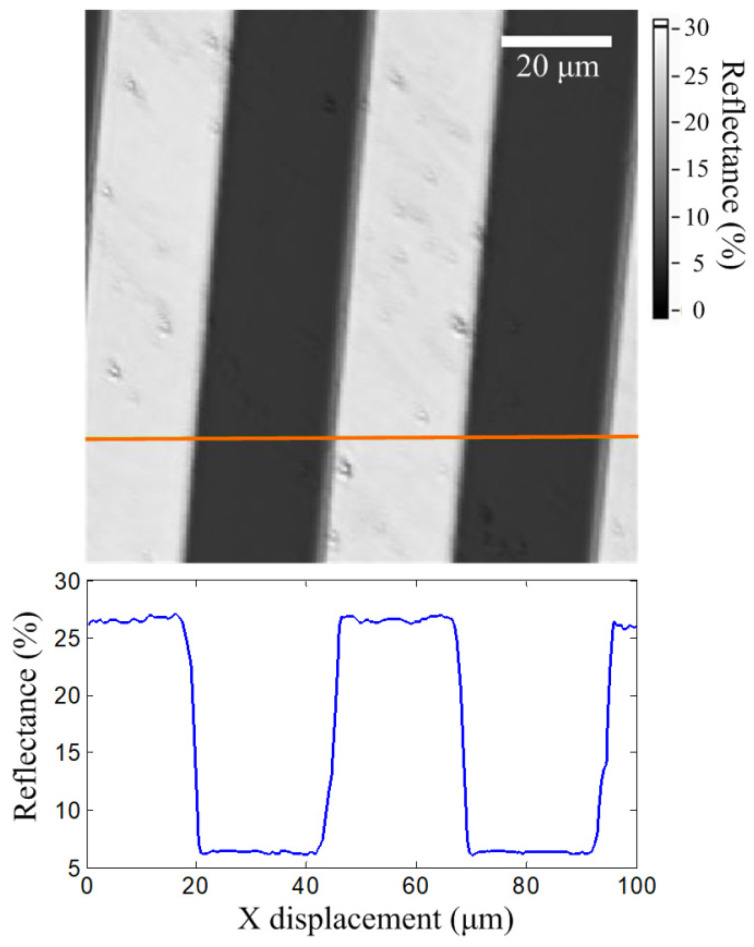
Real-time reflectance image on the grating R2L2S1N1.

A standard grating sample (R2L2S1N1, ThorLab, Newton, NJ, USA) was used for real-time reflectance imaging. The grating sample consisted of 120 nm thick chrome patterns on a glass substrate. The grating sample was also examined using a commercial reflection measurement system and the reflectances on chrome and glass surfaces were found to be 26.605% and 6.302%, respectively. After the grating sample was adjusted to the focal position of the pickup head, it was scanned using a closed-loop scanner in the XY plane. The scan range and rate were 100 µm × 100 µm (256 pixels × 256 pixels) and 0.2 line/s, respectively. The resonant scanner and lock-in amplifier were operated using the same settings as those described in Section 3.2. Here, we note that using a longer time constant of the lock-in amplifier can improve the reflectance resolution but limits the scan speed. The amplitude signal from the lock-in amplifier was captured by a programmable controller during the scanning. For real-time imaging, the amplitude signal was then transformed into reflectance by using (3). Figure 5 shows a reflectance image of the grating sample. The bright area with a higher reflectance of 26.53 ± 0.18% (mean ± standard deviation) was on the chrome layer and the dark area with a lower reflectance of 6.35 ± 0.08% was measured on the glass substrate. The average reflectances were consistent with the results of the commercial reflection measurement system, with a difference of less than 0.1%. Moreover, the image and standard deviation revealed that some surface particles and defects affected the uniformity of the reflectance, which cannot be observed by the single-point measurement of the commercial reflection measurement system. Furthermore, small particles with a diameter of approximately 1.5 µm can be resolved, which proves that the proposed system equips micrometer-scale resolution.

## 4. Conclusions

In this study, a novel method for real-time imaging of quantitative reflectance based on an astigmatic optical profilometer was proposed. By integrating a lock-in amplifier, the FES amplitude can be accurately measured and converted into an analog amplitude signal for real-time imaging. The relationships between the S-curve slope, FES amplitude, and reflectance were examined and the results proved that both the S-curve slope and FES amplitude were linearly proportional to the reflectance. By utilizing the fitting linear Equation (3), the amplitude signal can be converted into the reflectance in real time. The experimental results demonstrate that the reflectance of chrome and glass materials on a grating sample can be accurately measured. The measured reflectances were consistent with the results obtained by the commercial reflection measurement system, with a difference of less than 0.1%. The difference was below the standard deviation owing to the surface uniformity. The reflectance measurement is currently limited to the red-light wavelength. By adopting a Blu-ray pickup head, three wavelengths, 405, 650, and 780 nm, can be obtained [17].

## Figures and Tables

**Table 1:** Experimental results on the twelve optical filters with different reflectances.

Product Number	Reflectance (%)	Slope (V/μm)	Amplitude Signal (V)	FES Amplitude (V)
A3380020-4	7.995	0.0433	0.172	0.0241
A3380020-1	11.455	0.0847	0.345	0.0483
A3380020-2	18.714	0.1667	0.674	0.0944
A3380018-5	24.403	0.1751	0.805	0.1127
A3380020-5	37.510	0.3411	1.339	0.1875
A3380020-3	49.478	0.4735	1.836	0.2570
A3380018-010	50.873	0.4852	1.901	0.2661
A3380018-011	62.771	0.5738	2.256	0.3158
A3380018-3	72.776	0.6538	2.753	0.3854
A3380018-7	88.695	0.7861	3.323	0.4652
A3380018-8	91.922	0.8089	3.422	0.4791
A3380021-4	97.716	0.9578	3.740	0.5228

## Data Availability

Not applicable.
